# Psychosocial Predictors of Drop-Out from Organised Sport: A Prospective Study in Adolescent Soccer

**DOI:** 10.3390/ijerph192416585

**Published:** 2022-12-09

**Authors:** Jenny Back, Andreas Stenling, Bård Erlend Solstad, Petra Svedberg, Urban Johnson, Nikos Ntoumanis, Henrik Gustafsson, Andreas Ivarsson

**Affiliations:** 1 School of Health and Welfare, Halmstad University, P.O. Box 823, 301 18 Halmstad, Sweden; 2 Department of Psychology, Umeå University, 901 87 Umeå, Sweden; 3 Department of Sport Science and Physical Education, University of Agder, 4630 Kristiansand, Norway; 4 Norwegian Research Centre for Children and Youth Sports, 0806 Oslo, Norway; 5 Danish Centre for Motivation and Behaviour Science, Department of Sports Science and Clinical Biomechanics, University of Southern Denmark, 5230 Odense, Denmark; 6 Department of Educational Studies, Karlstad University, 651 88 Karlstad, Sweden; 7 Department of Sport and Social Sciences, Norwegian School of Sport Sciences, 0863 Oslo, Norway

**Keywords:** adolescents, drop-out, soccer, sport participation

## Abstract

In recent years an increased drop-out rate in adolescents’ soccer participation has been observed. Given the potentially adverse consequences of drop-out from soccer, more information about risk factors for drop-out is warranted. In the current study, Classification and Regression Tree (CRT) analysis was used to investigate demographic and motivational factors associated with an increased risk of drop-out from adolescent soccer. The results of this study indicate that older age, experiencing less autonomy support from the coach, less intrinsic motivation, being female, and lower socioeconomic status are factors associated with an increased risk of drop-out. An interpretation of the results of this study is that coaches play a central part in creating a sports context that facilitates motivation and continued soccer participation. Based on the findings of the current study we propose that soccer clubs implement theoretically informed coach education programs to help coaches adopt autonomy-supportive coaching strategies.

## 1. Introduction

Engaging in sport from an early age and staying active through adolescence can promote healthy lifestyle habits as well as physical and psychological health later in life [1,2]. Participation in organised sports during adolescence is, for instance, related to higher levels of physical activity [1], lower stress, and better coping with unexpected challenges and day-to-day demands in young adulthood [2]. Sports participation also has short-term physical (e.g., improved motor skills and aerobic fitness), psychological (e.g., fewer depressive symptoms and improved self-esteem), and social health-related (e.g., better social skills and lower social isolation) benefits [3,4,5]. Conversely, drop-out (i.e., disengagement from sports participation) may constitute a major health concern, as it is associated with reduced levels of physical activity, worse mental health, and reduced well-being [2,6,7].

Due to its social nature, team sport participation (e.g., soccer), compared to participation in, for instance, an individual sport, has been associated with additional benefits, such as lower levels of anxiety and depression [8,9], more social support, better self-esteem, and more social interaction [8,10]. Soccer is among the most popular sports, with 270 million participants worldwide [11]. It is also one of the major sports for adolescents worldwide [12]. Despite its popularity, approximately 25% of adolescents aged between 10 and 18 years drop out of soccer annually [12]. In Sweden, participation rates peak around the age of 12 and then drop consistently during the following youth years, with participant numbers decreasing in particular between the ages of 13–17 years [13]. This may, in part, be due to adolescents sampling different sports, but may also reflect dissatisfaction or negative experiences within the soccer context [14].

Given the potential adverse consequences of drop-out from soccer (and team sports in general), preventive programs to create team sports environments that support adolescents’ continued sports participation should be prioritized. However, to develop effective prevention programs, more knowledge about why adolescents drop out is needed [14,15,16].

Theoretical frameworks of factors that influence sports participation and drop-out, such as the social-ecological model of sport attrition [15], leisure constraints theory [16], and the process–person–context–time model [17] offer theoretical explanations for adolescents’ decisions to drop out from soccer. These models highlight a complex pattern of interactions between many different factors at various levels that influence drop-out (e.g., process; competition structure, relative age, person; sex, competence perception, context; parental socio-demographics, social support, and time; time-use characteristics, perceived time challenges).

In a review by Temple and Crane [14], several factors related to adolescents’ decisions to drop out of soccer were identified. Specifically, low perceived competence, lack of fulfilment of basic psychological needs, and poor relationships with teammates or coaches were among the most frequently listed in the included studies [14]. Similarly, a recent meta-analysis of factors associated with drop-out from adolescents’ team sports identified lack of fulfilment of basic psychological needs, lack of perceived social support from friends, family, and coaches, and lower levels of self-determined motivation as three of the factors that increased the risk for drop-out in adolescents’ team sports [18].

Previous research also indicate that demographic factors may influence drop-out from soccer and highlight, for instance, differences between age groups, boys and girls, and socioeconomic factors. Specifically, girls are more likely to drop out of soccer due to negative coaching experiences (e.g., yelling, not listening to players, lack of support) compared to boys [19]. Furthermore, children born later in the year are overrepresented among those who drop out of soccer [14]. Additionally, socioeconomic status (e.g., low household income and lower parental education) has been associated with drop-out [20].

As described above, both theoretical models of drop-out from sports among adolescents (e.g., the process–person–context–time model) and previous research on drop-out from soccer indicate that numerous factors may influence drop-out in adolescents’ soccer. Many of the previously described factors can, however, be attributed to motivation, and previous studies of drop-out and motivation highlighted the importance of the quality of motivation for persistence in both team and individual sports [21,22].

One of the most widely used theories of motivation in sports psychology [23] is self-determination theory (SDT) [24]. SDT is a theory of human motivation that describes behavioural regulations (e.g., why adolescents engage in sports), social factors (e.g., coach behaviours), and processes (i.e., need satisfaction and frustration) that either facilitate or undermine the quality of motivation. A fundamental tenet of SDT is that motivation can be classified along a self-determination continuum representing different levels of internalisation of the regulation of behaviour. This continuum ranges from amotivation to different types of extrinsic motivation to intrinsic motivation. Amotivation is described as a lack of motivation/intention for performing a particular behaviour, for instance, playing soccer, and is characterised by non-regulation. Controlled types of extrinsic motivation are (a) external motivation, such as playing soccer to obtain rewards or avoid punishment and (b) introjected motivation, defined as playing soccer to avoid guilt and shame and/or to attain ego enhancements and feelings of worth. Conversely, autonomous types of extrinsic motivation are (a) identified motivation and (b) integrated motivation, characterised by playing soccer because it is personally important and valued, or aligned with other aspects of the self, respectively. Finally, intrinsic motivation refers to engaging in an activity out of interest and inherent satisfaction, for example, playing soccer for the pleasure of participating in the activity. Intrinsic motivation reflects a will to play, explore, and develop one’s competencies and capacities. Accordingly, SDT differentiates between autonomous and controlled types of motivation based on the extent to which they represent autonomous or controlled regulations. While autonomous motivation stems from a sense of volition, controlled motivation comes from experiences of internal or external pressure.

Within SDT, the social environment is considered key to the development of these different types of motivation, behavioural persistence, and healthy functioning. Specifically, SDT posits that for humans to be optimally motivated, the social environment must support the fulfilment of three basic psychological needs, those for autonomy, competence, and relatedness [24]. These needs are satisfied when people feel a sense of volition regarding their choices and decisions in the context at hand (need for autonomy), effective and able to master tasks within their social environment (need for competence), and a sense of connection to and belongingness with others in a particular context (need for relatedness).

One aspect of the social environment suggested to influence need satisfaction is autonomy support. In soccer, the coach plays a significant part in providing autonomy support and shaping athletes’ experiences [25]. An autonomy-supportive sports context (e.g., where coaches offer athletes choices, provide them with rationales, and include athletes in decision processes) facilitates the fulfilment of basic psychological needs and promote autonomous and intrinsic motivation. More specifically, coaches that act in an autonomy-supportive manner can contribute to psychological need satisfaction and, in turn, adaptive forms of motivation (i.e., autonomous and intrinsic motivation) that is associated with positive athlete outcomes (e.g., increased persistence, improved performance, well-being). In practice, autonomy supportive coaching may include allowing athletes to choose between different training activities, explaining the advantages of a skill to give athletes an understanding of why it is practiced, and focusing on self-evaluative criteria of performance [26]. Moreover, although all three of the basic psychological needs are considered equally important to human development and well-being, autonomy support plays a critical role, not only to facilitate autonomy, but the needs for competence and relatedness too. This is because support for autonomy makes people more able to seek out and find satisfaction for the needs for competence and relatedness as well [24]. For instance, a recent review of research on autonomy support in sport and exercise settings found strong evidence for positive associations between autonomy support and athlete basic psychological needs for autonomy, competence, and relatedness [25]. Conversely, a controlling context (e.g., where athletes are pressured to behave, feel, and think in specific ways) frustrates fulfilment of basic psychological needs and therefore undermines autonomous and intrinsic motivation [24]. Thus, coaches’ behaviours (i.e., autonomy-supportive vs. controlling), psychological need satisfaction, and different qualities of motivation appear to be key predictors of continued sports participation in young athletes [27]. A limitation in previous research on drop-out from soccer among adolescents is that relatively few prospective studies have been conducted [28]. Furthermore, the prospective studies conducted have, in many cases, applied a reductionist approach where each potential risk factor has been analysed separately using, for example, difference tests (e.g., *t*-test) [14]. A reductionist approach provides a very limited picture of why adolescents drop out of sports and most theories/models (e.g., the social-ecological model of sport attrition [15], leisure constraint theory [16], process–person–context–time model [17]) highlight that drop-out is influenced by complex interactions between several factors. Moreover, soccer is, in terms of the number of participants, the major sport worldwide [11]. High drop-out rates therefore represent millions of adolescents leaving the sport. With this in mind and considering the potential adverse consequences of leaving soccer (and sports in general), knowledge that can inform initiatives to prevent drop-out from soccer is important. In the current study, we therefore aimed to overcome limitations in previous research and investigate how combinations of multiple demographic and motivational factors are simultaneously associated with the risk of drop-out in adolescent soccer players using a prospective design.

## 2. Materials and Methods

### 2.1. Participants

Participants were 738 adolescent soccer players (462 males and 275 females) from 16 soccer clubs in Sweden. Participants were between 11 and 17 years old (M = 13.72, SD = 1.77). The participants had, on average, participated in soccer for 7.56 years (SD = 2.74) and practiced 4.85 (SD = 2.36) hours per week. Approximately half of the participants (52.1%) were engaged in at least one more organised sport apart from soccer. A total of 159 participants (21.5%) had experienced a serious injury with more than one month of rehabilitation during the previous season. In general, the participants reported high socioeconomic status (M = 4.39, SD = 0.74) as reported on a five-point Likert scale between 1 (not at all well-off) and 5 (very well-off).

### 2.2. Measures

#### 2.2.1. Demographic Data, Socioeconomic Status, Training, and Injury History

We collected demographic data such as soccer club membership, gender, age (year, month), years in soccer, and the number of additional sports (except soccer). Additionally, the players were asked to indicate if they, during the previous season, had experienced an injury with more than one month’s rehabilitation. Participants’ socioeconomic status was measured using a single question (“How would you describe the economic situation in your family?”), asking them to indicate, on a five-point Likert scale between 1 (not at all well-off) and 5 (very well-off), how well-off, financially, they think their family is. This item is easily understood and has been used in previous research [29].

#### 2.2.2. Motivation

A Swedish version of the Behavioural Regulation in Sport Questionnaire [30,31] (BRSQ) was used to assess the different types of motivation according to SDT. Participants are asked to indicate how well the items corresponded to their reasons for participating in soccer, responding on a seven-point Likert scale from 1 “not at all true” to 7 “very true”. We included five four-item subscales designed to measure amotivation (e.g., “I participate in my sport, but I question why I continue”, McDonald’s ω = 0.61), external regulation (e.g., “I participate in my sport in order to satisfy people who want me to play”, McDonald’s ω = 0.68), introjected regulation (e.g., “I participate in my sport because I would feel like a failure if I quit”, McDonald’s ω = 0.69), identified regulation (e.g., “I participate in my sport because I value the benefits of my sport”, McDonald’s ω = 0.71), and intrinsic motivation (e.g., “I participate in my sport because I enjoy it”, McDonald’s ω = 0.77).

#### 2.2.3. Coach Autonomy Support

A Swedish version of the short six-item Sport Climate Questionnaire [32] (SCQ) was used to measure participants’ perception of autonomy support from their coach.

Participants are asked to rate on a seven-point Likert scale, ranging from 1“strongly disagree” to 7 “strongly agree”, how they feel about their experiences with their coach (e.g., “I feel my coach provides me choices and options”, McDonald’s ω = 0.87).

#### 2.2.4. Drop-Out from Soccer

Information about drop-out from soccer was collected from coaches of participating clubs during the following three seasons after the above measures were collected. We created a list with the names of the participants in the study. One year after the data were collected, this list was sent by mail to responsible coaches for each participating team. The coaches were asked to indicate players who had terminated their participation in the club. The list was then sent back to the researchers.

### 2.3. Procedure

In collaboration with several Regional Soccer Associations, a stratified sample of Swedish soccer clubs with adolescent female and male players was recruited for the present study. Initially, directors of soccer clubs were contacted with information about the overall aim of the study. Following the agreement to take part in the study, an information meeting where study procedures were outlined was scheduled with coaches. After this initial meeting, information about the project was distributed to the parents of the adolescents. Because participants below 15 years of age were to take part in the study, both guardians and the child had to agree to be involved in the study. Thus, we first asked parents to sign informed consent agreeing that their adolescent may take part in the study. Then, all youth who received informed consent from their parents were asked to provide informed consent prior to the first data collection. During the following three seasons, coaches of participating clubs were contacted and asked to confirm which players had stopped playing soccer. The study was approved by the national ethical review board (Nr: 2019-01643).

### 2.4. Analyses

Classification and Regression Tree (CRT) analysis was used to investigate combinations of demographic and motivational factors that may predict drop-out from adolescent soccer. The CRT analysis investigates the relationship between predictors (in the present study: behavioural regulation, coach autonomy support, age, gender, socioeconomic status, number of other sports, and injury in previous season) and the outcome variable (in the present study: drop-out from soccer) by searching for combinations of predictors that best explain the outcome variable [33].

With CRT analysis, a hierarchical and graphical representation of interactions between variables in the shape of a tree diagram is produced. The data are classified into subgroups (or nodes) according to the variable that best explains the dependent variable. A node that is divided into sub-nodes (i.e., child nodes) is called a parent node. Terminal nodes are nodes that do not split into further nodes. Nodes are split into sub-nodes that maximize within-node homogeneity and between-node heterogeneity. The extent to which a node does not represent a homogenous subset of cases is called impurity. Each sub-node continues to generate more sub-nodes based on the strongest predictor until a stopping rule of minimum change in improvement triggers. In CRT, “splitting stops when the relative reduction in error resulting from the best split falls below a pre-specified threshold”. Typical values of this threshold are in the range of 0.001–0.05 [34]. This is the minimum decrease in impurity required to split a node. We followed the criteria suggested by Machuca et al. [33] for the analysis.

More specifically, the criteria were that (a) the minimum number of cases in the parent node = 70, and (b) the minimum of cases in the terminal nodes = 35. For data with a small number of cases, higher criteria values may result in trees with no nodes below the root node; in this case, lowering the minimum values may produce more useful results [35].

We applied tree pruning to avoid overfitting, with a maximum acceptable difference in risk between the pruned tree and the subtree of one standard error. Tree pruning means the tree is grown (i.e., generate more sub-nodes) until the stopping criteria are met, and then it is trimmed automatically to the smallest subtree based on the specified maximum difference in risk [35].

To validate the tree, we applied the tenfold cross-validation application. With tenfold cross-validation the dataset is first randomly partitioned into ten roughly equal parts. Next, nine parts of the data are used to create the largest tree possible and the remaining part (1/10) of the data is used to validate this tree. The process is repeated ten times using different combinations of the data. The results of the ten tests are combined to calculate error rates for trees of each possible size and are then applied to prune the full tree [33].

We treated missing data by surrogated splits. When using surrogated splits, other variables that have a high association with the original variable are used for classification in the cases where the value for the original variable is missing [35].

We calculated risk differences (RD, with corresponding 95% confidence intervals (CI)) to illustrate the magnitude of difference in proportion of drop-out players between the subgroups. All analyses were conducted in the IBM SPSS Statistics version 28 software (SPSS Inc., Chicago, IL, USA).

## 3. Results

### 3.1. Descriptive Statistics

Of the participants, 256 (34.7%) dropped out from soccer until the end of the 2021 season. The participants reported, in general, high levels of autonomous behavioural regulations (i.e., intrinsic and identified) and lower levels of controlled regulations as well as amotivation. Additionally, they reported high values of perceived autonomy support from the coach. Descriptive statistics and correlations between the study variables are presented in Table 1.

### 3.2. Risk Factors for Drop-Out

**Figure 1:**
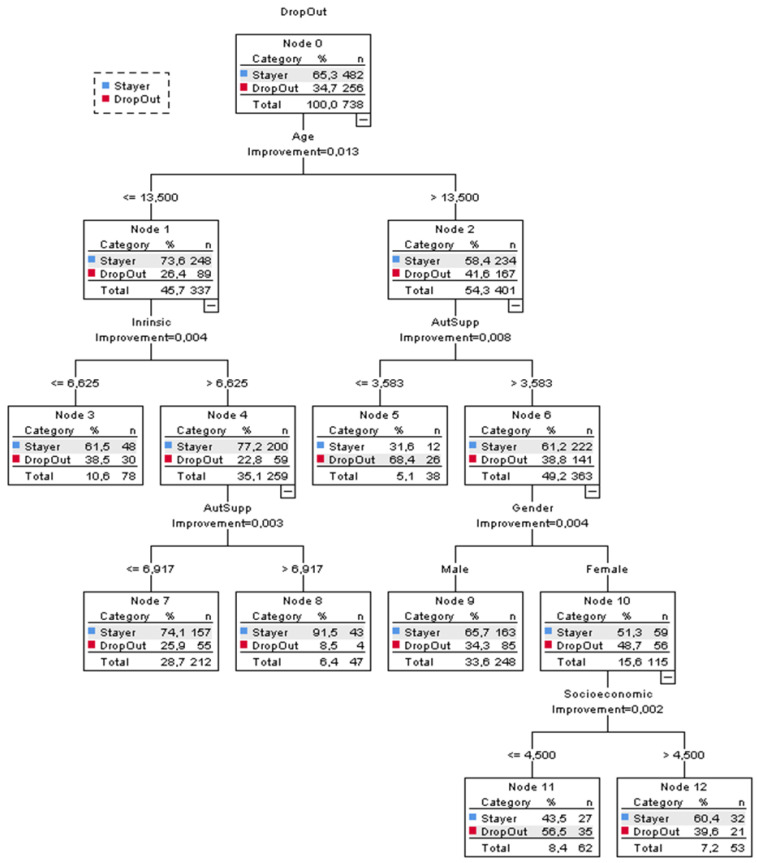
Decision tree of demographic and motivational risk factors for drop-out from soccer.

Results of the CRT analysis are presented in Figure 1. At the top of the tree, age was identified as the most important predictor of drop-out. Specifically, older players (>13.5 years) were exposed to an increased risk of drop-out compared to younger players (RD = 15.2%, 95% CI = (8.50, 21.98)). For the older players, experiencing lower levels of autonomy support from the coach was the most important risk factor of drop-out (RD = 29.6%, 95% CI = (13.97, 45.19)). At the next level of the tree, gender was identified as an additional risk factor of drop-out. More specifically, among the players who experienced higher levels of autonomy support from their coach, being female was associated with an increased risk of drop-out (RD = 14.4%, 95% CI = (3.69, 25.43)). For females, lower socioeconomic status had a relation with drop-out (RD = 16.9%, 95% CI = (−1.22, 34.88)). For the younger players (≤13.5), experiencing less intrinsic motivation was associated with an increased risk of drop-out (RD = 15.7%, 95% CI = (3.74, 27.63)). Among the younger players with higher levels of intrinsic motivation, experiencing less autonomy support from the coach was related to increased risk of drop-out (RD = 17.4%, 95% CI = (7.51, 27.36)).

## 4. Discussion

The aim of the current study was to investigate how combinations of multiple demographic and motivational factors were simultaneously associated with the risk of drop-out in adolescent soccer players. The CRT analysis showed that older age, experiencing less autonomy support from the coach, less intrinsic motivation, being female, and lower socioeconomic status were all associated with an increased risk of drop-out.

In the current study, older player age (i.e., 13.5 years or more) was identified as the strongest risk factor for drop-out. A potential explanation may be that, with age, competing interests (e.g., friends and social events) and demands from other activities (e.g., school) increase, and some adolescents might, therefore, prioritize other activities. In line with this reasoning, time-related factors (e.g., competing demands for time) have been identified as some of the most frequently reported reasons for drop-out from soccer among adolescents [14]. Similarly, lack of time (e.g., due to increased schoolwork) and other interests have been found to be important factors related to drop-out in other team sports (e.g., handball [36], floorball [37]). In addition, demands within a sport may become higher with age. For example, previous studies indicated that athletes experience sport becoming increasingly performance- and result-oriented with age [37], which may influence the quality of motivation and sport participation negatively [38,39]. Additionally, the increased focus on performance will also increase the number of players who will be de-selected from the team [40,41].

In combination with older age, experiencing lower levels of autonomy support from the coach was associated with an increased risk of drop-out from soccer. Within SDT, the importance of the social environment for behavioural persistence and motivational quality is highlighted. Accordingly, perception of an autonomy-supportive sport context facilitates motivation that is more intrinsically regulated, while perception of a controlling context undermines intrinsic motivation and behaviour adherence [24]. From an SDT perspective, a potential explanation to the results of this study may be that lower levels of autonomy support, over time, might affect motivational quality, something that in turn may be related to future drop-out [42].

Among the players that experienced higher levels of autonomy support, females were more likely to drop out. Moreover, the combination of being female and having lower socioeconomic status was associated with an increased risk for drop-out from soccer. Because the number of girls’ teams in Sweden decreases when players grow older, caregivers and players may need to invest more time and money and travel greater distances to be able to practice and compete in soccer. As an additional explanation of the relation between socioeconomic status and drop-out, it could be argued that the recognition of sports participation as an important activity may be a component of socioeconomic status [43]. It is possible that in the families with lower socioeconomic status there may not be a strong sense of the importance of sports participation and the resources to prioritize and make the required investments of time and social commitment may not be available. This line of reasoning may be supported by previous research that indicate that adolescents from affluent families tend to participate more often in organised sports [44]. Moreover, adolescents from families with less resources often highlight discouragement from parents as important to their decision to drop out from sport [45].

While perceived autonomy support was the strongest risk factor of drop-out for the older adolescents, experiencing less intrinsic motivation was the most important factor associated with drop-out among the younger players (i.e., 13.5 years or younger). Although both the younger players that continued in soccer and dropouts experienced high levels of intrinsic motivation in general, in our study, even a small reduction in intrinsic motivation was identified as a risk factor for drop-out. The result substantiates the importance of intrinsic motivation in soccer participation. Interestingly, in our study, experiencing less intrinsic motivation was identified as a risk factor for drop-out only among the younger adolescents. Similarly, previous research has reported age-related differences in both youth sport motivation and barriers to participation [46,47,48]. It is possible that when sport contexts and the demands placed on athletes inside and outside sport change as players grow older (e.g., more focus on performance in sport, increased schoolwork), the relation between demographic factors, motivational factors, and drop-out become more complex. In the younger ages, however, perceiving soccer as interesting, fun, and enjoyable may be the most important predictors of soccer continuation/drop-out.

### Strengths, Limitations, and Future Directions

The prospective design and analytical method are strengths of this study. First, a prospective design where potential predictors are measured prior to the outcome strengthens the credibility of conclusions drawn about the causal relationships between study variables (i.e., that risk factors precede the outcome). Moreover, predictors of dropout behaviour do not act independently of each other (e.g., [17]). Therefore, using an analytical method that investigates how combinations of risk factors simultaneously interact to predict drop-out from soccer additionally strengthens the study. Although these are important strengths, weaknesses are also present. First, we did not measure controlling coach behaviours. Coach behaviours are essential elements of the sport context, and more positive (i.e., autonomy-supportive) as well as negative (i.e., controlling) coach behaviours may affect the relation between sport context, motivational quality, and drop-out from soccer among adolescents [49]. Second, we did not ask the players that dropped out about the reasons why they did so, and some might have transferred to another sport. Delineating sampling of sporting activities and permanent drop-out may be important in future studies. Although players that have already dropped out may be less inclined to answer questions regarding their soccer participation [50], future studies could also benefit from obtaining more in-depth information about the underlying reasons and interactions between different reasons for dropping out from sport from the adolescents’ own perspective. 

## 5. Conclusions

The results of this study illustrate that the risk of drop-out from youth soccer is influenced by combinations of demographic and motivational factors. Moreover, different combinations of risk factors are more important depending on the age and gender of the athletes.

An interpretation of the results of this study is that coaches play a central part in creating a sports context that facilitates motivation and continued soccer participation. Practically, this means that coaches should focus on promoting a high-quality motivational climate, for instance by implementing an autonomy-supportive coaching strategy, making the activity feel more important, and supporting young athletes’ feelings of autonomy, competence, and relatedness. However, in the current study, females were, in comparison to males, exposed to an increased risk of drop-out when experiencing higher levels of autonomy support. A conclusion drawn from these results is that although autonomy support from the coach is prominent, in female soccer important risk factors for drop-out may also be related to contextual factors such as socio-economy and the availability of soccer teams.

To conclude, the results of this study show that, except for age, the factors identified as most important to the risk of drop-out from adolescent soccer are modifiable variables related to athletes’ motivation. Moreover, the findings of the current study indicate that soccer clubs would benefit from implementing theoretically informed coach education programs to help coaches adopt autonomy-supportive coaching strategies that may facilitate autonomous and intrinsic motivation.

## Figures and Tables

**Table 1:** Descriptive statistics and correlations between the study variables.

Variable	M (SD)	Age	HT	SE	IN	ID	IJ	EXT	AMOT	AS
1. Age	13.72 (1.77)	—								
2. HT	4.85 (2.36)	0.49 *	—							
3. SE	4.39 (0.73)	−0.15 *	−0.06	—						
4. IN	6.54 (0.91)	−0.05	−0.09 *	0.05	—					
5. ID	4.70 (1.48)	−0.20 *	−0.11 *	0.15 *	0.34 *	—				
6. IJ	1.60 (0.95)	0.12	0.09 *	−0.12 *	−0.39 *	−0.05	—			
7. EXT	1.49 (0.86)	0.12 *	0.12 *	−0.05	−0.50 *	−0.11 *	0.62 *	—		
8. AMOT	1.37 (0.71)	0.07	0.01	−0.03	−0.54 *	−0.18 *	0.58 *	0.56 *	—	
9. AS	5.58 (1.14)	−0.21 *	−0.11 *	0.17 *	0.23 *	0.23 *	−0.23 *	−0.18 *	−0.18 *	—

HT = hours training per week; SE = socioeconomic status; IN = intrinsic regulation; ID = identified regulation; IJ = introjected regulation; EXT = external regulation; AMOT = amotivation; AS = coach autonomy support; * = *p* < 0.05.

## Data Availability

The data presented in this study are available on request from the corresponding author.

## References

[1] Kjonniksen L., Anderssen N., Wold B. (2009). Organized youth sport as a predictor of physical activity in adulthood. Scand. J. Med. Sci. Sports.

[2] Murray R.M., Sabiston C.M., Dore I., Belanger M., O’Loughlin J.L. (2021). Association between pattern of team sport participation from adolescence to young adulthood and mental health. Scand. J. Med. Sci. Sports.

[3] Andersen M.H., Ottesen L., Thing L.F. (2019). The social and psychological health outcomes of team sport participation in adults: An integrative review of research. Scand. J. Public Health.

[4] Eime R.M., Young J.A., Harvey J.T., Charity M.J., Payne W.R. (2013). A systematic review of the psychological and social benefits of participation in sport for adults: Informing development of a conceptual model of health through sport. Int. J. Behav. Nutr. Phys. Act..

[5] Krustrup P., Dvorak J., Junge A., Bangsbo J. (2010). Executive summary: The health and fitness benefits of regular participation in small-sided football games. Scand. J. Med. Sci. Sport..

[6] Vella S.A.P., Cliff D.P.P., Magee C.A.P., Okely A.D.E. (2014). Sports Participation and Parent-Reported Health-Related Quality of Life in Children: Longitudinal Associations. J. Pediatr..

[7] Vella S.A., Cliff D.P., Magee C.A., Okely A.D. (2015). Associations between sports participation and psychological difficulties during childhood: A two-year follow up. J. Sci. Med. Sport.

[8] Evans M.B., Allan V., Erickson K., Martin L.J., Budziszewski R., Côté J. (2017). Are all sport activities equal? A systematic review of how youth psychosocial experiences vary across differing sport activities. Br. J. Sport. Med..

[9] Pluhar E., McCracken C., Griffith K.L., Christino M.A., Sugimoto D., Meehan W.P. (2019). Team Sport Athletes May Be Less Likely to Suffer Anxiety or Depression than Individual Sport Athletes. J. Sports Sci. Med..

[10] Zuckerman S.L., Tang A.R., Richard K.E., Grisham C.J., Kuhn A.W., Bonfield C.M., Yengo-Kahn A.M. (2021). The behavioral, psychological, and social impacts of team sports: A systematic review and meta-analysis. Physician Sportsmed..

[11] FIFA (2006). FIFA Big Count 2006: 270 Million People Active in Football. https://resources.fifa.com/image/upload/big-count-estadisticas-520058.pdf?cloudif=mzid0qmguixkcmruvema.

[12] Møllerløkken N.E., Lorås H., Pedersen A.V. (2015). A Systematic Review and Meta-Analysis of Dropout Rates in Youth Soccer. Percept. Mot. Ski..

[13] (2021). RF Idrottsrörelsen i Siffror. https://www.rf.se/download/18.407871d3183abb2a6131d8f/1665068212883/2021%20Idrotten%20i%20siffror%20-%20RF.pdf.

[14] Temple V.A., Crane J.R. (2016). A systematic review of drop-out from organized soccer among children and adolescents. Soccer Soc..

[15] Balish S.M., McLaren C., Rainham D., Blanchard C. (2014). Correlates of youth sport attrition: A review and future directions. Psychol. Sport Exerc..

[16] Crane J., Temple V. (2014). A systematic review of dropout from organized sport among children and youth. Eur. Phys. Educ. Rev..

[17] Moulds K., Galloway S., Abbott S., Cobley S.P. (2022). Youth sport dropout according to the Process-Person-Context-Time model: A systematic review. Int. Rev. Sport Exerc. Psychol..

[18] Back J., Johnson U., Svedberg P., McCall A., Ivarsson A. (2022). Drop-out from team sport among adolescents: A systematic review and meta-analysis of prospective studies. Psychol. Sport Exerc..

[19] Keathley K., Himelein M.J., Srigley G. (2013). Youth soccer participation and withdrawal: Gender similarities and differences. J. Sport Behav..

[20] Vella S.A., Cliff D.P., Okely A.D. (2014). Socio-ecological predictors of participation and dropout in organised sports during childhood. Int. J. Behav. Nutr. Phys. Act..

[21] Jõesaar H., Hein V., Hagger M.S. (2011). Peer influence on young athletes’ need satisfaction, intrinsic motivation and persistence in sport: A 12-month prospective study. Psychol. Sport Exerc..

[22] Pelletier L.G., Fortier M.S., Vallerand R.J., Brière N.M. (2001). Associations Among Perceived Autonomy Support, Forms of Self-Regulation, and Persistence: A Prospective Study. Motiv. Emot..

[23] Lindahl J., Stenling A., Lindwall M., Colliander C. (2015). Trends and knowledge base in sport and exercise psychology research: A bibliometric review study. Int. Rev. Sport Exerc. Psychol..

[24] Ryan R.M., Deci E.L. (2017). Self-Determination Theory: Basic Psychological Needs in Motivation, Development, and Wellness.

[25] Mossman L.H., Slemp G.R., Lewis K.J., Colla R.H., O’Halloran P. (2022). Autonomy support in sport and exercise settings: A systematic review and meta-analysis. Int. Rev. Sport Exerc. Psychol..

[26] Occhino J.L., Mallett C.J., Rynne S.B., Carlisle K.N. (2014). Autonomy-supportive pedagogical approach to sports coaching: Research, challenges and opportunities. Int. J. Sport. Sci. Coach..

[27] Quested E., Ntoumanis N., Viladrich C., Haug E., Ommundsen Y., Van Hoye A., Mercé J., Hall H.K., Zourbanos N., Duda J.L. (2013). Intentions to drop-out of youth soccer: A test of the basic needs theory among European youth from five countries. Int. J. Sport Exerc. Psychol..

[28] Van Yperen N.W., Jonker L., Verbeek J. (2022). Predicting Dropout from Organized Football: A Prospective 4-Year Study Among Adolescent and Young Adult Football Players. Front. Sport. Act. Living.

[29] Quon E.C., McGrath J.J. (2014). Subjective Socioeconomic Status and Adolescent Health: A Meta-Analysis. Health Psychol..

[30] Stenling A., Ivarsson A., Lindwall M., Gucciardi D.F. (2018). Exploring longitudinal measurement invariance and the continuum hypothesis in the Swedish version of the Behavioral Regulation in Sport Questionnaire (BRSQ): An exploratory structural equation modeling approach. Psychol. Sport Exerc..

[31] Lonsdale C., Hodge K., Rose E.A. (2008). The Behavioral Regulation in Sport Questionnaire (BRSQ): Instrument Development and Initial Validity Evidence. J. Sport Exerc. Psychol..

[32] Hagger M.S., Chatzisarantis N.L.D., Hein V., Pihu M., Soós I., Karsai I. (2007). The perceived autonomy support scale for exercise settings (PASSES): Development, validity, and cross-cultural invariance in young people. Psychol. Sport Exerc..

[33] Machuca C., Vettore M.V., Krasuska M., Baker S.R., Robinson P.G. (2017). Using classification and regression tree modelling to investigate response shift patterns in dentine hypersensitivity. BMC Med. Res. Methodol..

[34] Venkatasubramaniam A., Wolfson J., Mitchell N., Barnes T., JaKa M., French S. (2017). Decision trees in epidemiological research. Emerg. Themes Epidemiol..

[35] IBM IBM SPSS Decision Trees 28. https://www.ibm.com/docs/en/SSLVMB_28.0.0/pdf/IBM_SPSS_Decision_Trees.pdf.

[36] Bentzen M., Hordvik M., Stenersen M.H., Solstad B.E. (2021). A longitudinal transitional perspective on why adolescents choose to quit organized sport in Norway. Psychol. Sport Exerc..

[37] Eliasson I., Johansson A. (2020). The disengagement process among young athletes when withdrawing from sport: A new research approach. Int. Rev. Sociol. Sport.

[38] Gómez-López M., Merino-Barrero J.A., Manzano-Sánchez D., Valero-Valenzuela A. (2019). A cluster analysis of high-performance handball players’ perceived motivational climate: Implications on motivation, implicit beliefs of ability and intention to be physically active. Int. J. Sport. Sci. Coach..

[39] Russell W. (2021). An Examination of Sport Motivation, Motivational Climate, and Athlete Burnout within the Developmental Model of Sport Participation. J. Amat. Sport.

[40] Baker J., Wilson S., Johnston K., Dehghansai N., Koenigsberg A., de Vegt S., Wattie N. (2020). Talent Research in Sport 1990–2018: A Scoping Review. Front. Psychol..

[41] Johnston K., Wattie N., Schorer J., Baker J. (2018). Talent Identification in Sport: A Systematic Review. Sport. Med..

[42] Ntoumanis N. (2012). A self-determination theory perspective on motivation in sport and physical education: Current trends and possible future research directions. Motiv. Sport Exerc..

[43] Toftegaard-Støckel J., Nielsen G.A., Ibsen B., Andersen L.B. (2011). Parental, socio and cultural factors associated with adolescents’ sports participation in four Danish municipalities. Scand. J. Med. Sci. Sport..

[44] Hobza V., Maracek M., Hamrik Z. (2022). Organized Sport Activities of 11 to 15-Year-Old Adolescents: Trends from 2010–2018 and Socioeconomic Context. Int. J. Environ. Res. Public Health.

[45] Espedalen L.E., Seippel Ø. (2022). Dropout and social inequality: Young people’s reasons for leaving organized sports. Ann. Leis. Res..

[46] Butt J., Weinberg R.S., Breckon J.D., Claytor R.P. (2011). Adolescent Physical Activity Participation and Motivational Determinants Across Gender, Age, and Race. J. Phys. Act. Health.

[47] Sevil-Serrano J., Aibar A., Abós Á., Generelo E., García-González L. (2022). Improving motivation for physical activity and physical education through a school-based intervention. J. Exp. Educ..

[48] Wendling E., Flaherty M., Sagas M., Kaplanidou K. (2018). Youth athletes’ sustained involvement in elite sport: An exploratory examination of elements affecting their athletic participation. Int. J. Sport. Sci. Coach..

[49] Bartholomew K.J., Ntoumanis N., Thøgersen-Ntoumani C. (2009). A review of controlling motivational strategies from a self-determination theory perspective: Implications for sports coaches. Int. Rev. Sport Exerc. Psychol..

[50] Deelen I., Ettema D., Kamphuis C.B.M. (2018). Time-use and environmental determinants of dropout from organized youth football and tennis. BMC Public Health.

